# Natural Hendra Virus Infections in Captive Australian Black Flying Foxes, Queensland, Australia

**DOI:** 10.3201/eid3203.251350

**Published:** 2026-03

**Authors:** Victoria Boyd, Anjana Karawita, Jianning Wang, Shawn Todd, Sarah Riddell, Rachel Layton, Grace Taylor, Michael L. Kelly, Teegan Allen, Sarah Caruso, Christopher C. Broder, Richard J. Ploeg, Gough G. Au, Gary Crameri, Anthony W. Purcell, Michelle L. Baker

**Affiliations:** Commonwealth Scientific and Industrial Research Organisation, Health and Biosecurity, Geelong, Victoria, Australia (V. Boyd, S. Todd, S. Caruso, M.L. Baker); Commonwealth Scientific and Industrial Research Organisation Australian Animal Health Laboratory, Geelong (A. Karawita, J. Wang, S. Riddell, R. Layton, G. Taylor, M.L. Kelly, T. Allen, R.J. Ploeg, G.G. Au); Uniformed Services University, Bethesda, Maryland, USA (C.C. Broder); Monash University, Clayton, Victoria, Australia (A.W. Purcell)

**Keywords:** Hendra virus, viruses, zoonoses, bats, flying fox, serology, viral infection, recrudescence, Australia

## Abstract

We provide evidence for natural Hendra virus infections and associated serology in a cohort of Australian black flying foxes (*Pteropus alecto*) transferred from Queensland to the Australian Centre for Disease Preparedness in Victoria, Australia. This study supports the likelihood that flying foxes undergo cycles of infection and reinfection and possibly recrudescence.

Bats, including flying foxes, are natural reservoirs for a variety of viruses, many highly pathogenic in other species, including the henipaviruses Hendra virus (HeV) and Nipah virus (NiV). In Australia, HeV antibodies have been detected in all 4 species of Australian flying foxes; however, the Australian black flying fox (*Pteropus alecto*) is the primary reservoir for the original HeV genotype 1 variant (HeVg1) (*1*,*2*). Spillover events occurring annually from flying foxes into horses pose a potential risk for subsequent transmission to humans (*1*,*2*).

Government biosecurity authorities in Australia have recorded 90 outbreaks of HeV since 1994, when the virus was first identified, with HeV spillover events occurring predominantly during winter (June–August) (*2*). Research has also implicated nutrition and life history events, including the mating and birthing seasons, as imparting a higher risk of infection in Australian flying foxes (*3*). More recent findings have identified such environmental factors as habitat loss, droughts, and the scarcity of winter-flowering plants as implicit in driving bats to relocate to agricultural and urban areas. In those settings, bats feed on suboptimal foods that are often in close proximity to livestock, increasing the potential for spillover. In contrast, an abundance of winter flowers, which alleviate nutritional stress, appears to have a protective effect against HeV spillover (*4*).

We describe changes in HeV serology and infection status among a cohort of 20 *P. alecto* bats held in captivity in Queensland, Australia, and transported to The Australian Centre for Disease Preparedness (ACDP) in Victoria, Australia. This study was approved by the ACDP animal ethics committee (ACDP22004).

## The Study

**Figure 1:**
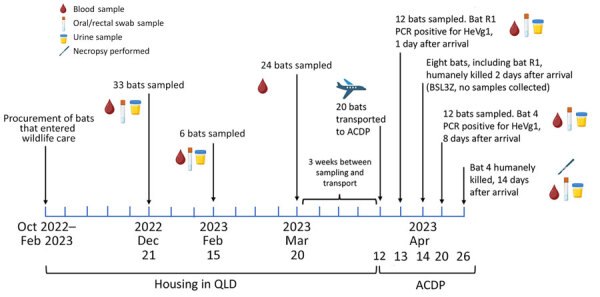
Timeline of events from inclusion of flying foxes in Queensland through to housing at ACDP from study of natural HeV infections in captive Australian black flying foxes, Queensland, Australia. Bats were added to the study as they became available December 2022–February 2023 after entering the care of bat rehabilitators in Queensland due to injuries. Samples collected from bat 4 at necropsy day 14 after arrival at ACDP included lung, liver, kidney, spleen, heart, ileum, large intestine, jejunum, salivary gland, retropharyngeal lymph node, submandibular lymph node, gonad, brain, cerebellum, urine, plasma, nasal wash, and oral and rectal swabs. Samples collected from individual bats are indicated on the timeline and included blood samples for serology and oral/rectal swab and urine samples for PCR. Urine was collected underneath individual cages for PCR testing on all days during the housing period at ACDP. ACDP, Australian Centre for Disease Preparedness; BSL, Biosafety Level; HeV, Hendra virus; HeVg1, HeV genotype 1 variant.

We included in this study flying foxes submitted to bat rehabilitators because of minor injuries that were otherwise healthy but nonreleasable. The cohort was selected from 39 bats housed in outdoor rehabilitation enclosures in Queensland, separate from other bats and screened by an in-house Luminex indirect antibody assay as described previously (*5*) (Appendix Table). We selected 20 bats with the lowest HeV serostatus and best overall condition for transport (12 seronegative, 8 low-positive). We charted the age and sex of the cohort (Table), which included 15 adults (6 female, 9 male), 4 subadults (2 female, 2 male), and 1 juvenile male bat. Tests revealed no viral RNA for known zoonotic viruses (HeVg1, HeV genotype 2 variant, Menangle virus, and Australian bat lyssavirus) in swab or urine specimens before transport, and HeV serostatus remained stable over 3 sampling events for up to 6 months in captivity in Queensland. Three weeks after the final Queensland testing (April 2023), we transported the animals to ACDP in Victoria (Figure 1).

**Table:** Sex, age, and location of origin of 20 bats included in study examining Hendra virus infections in captive Australian black flying foxes, Queensland, Australia*

Flying fox ID	Sex	Age	Location of origin	Housing at ACDP
1	M	Adult	Beaudesert	BSL3Z
2	M	Subadult	Beaudesert	BSL3Z
3	F	Adult	Beaudesert	BSL4
4	M	Adult	Beaudesert	BSL4
5	M	Adult	Logan Reserve	BSL4
6	M	Adult	Beaudesert	BSL4
7	M	Adult	Beaudesert	BSL4
8	F	Subadult	Beaudesert	BSL4
9	F	Adult	Beaudesert	BSL3Z
10	M	Adult	Beaudesert	BSL3Z
11	F	Adult	Logan Reserve	BSL4
12	M	Subadult	Beaudesert	BSL4
13	F	Subadult	Beaudesert	BSL4
14	M	Adult	Beaudesert	BSL4
15	F	Adult	Logan Reserve	BSL4
16	M	Adult	Beaudesert	BSL4
R-1	M	Adult	Beaudesert	BSL3Z
R-2	M	Juvenile	Beaudesert	BSL3Z
R-3	F	Adult	Beaudesert	BSL3Z
R-4	F	Adult	Beaudesert	BSL3Z

*After arrival at ACDP, flying foxes were either housed in a group aviary at BSL3Z or in individual cages in a BSL4 room. ACDP, Australian Centre for Disease Preparedness; BSL, Biosafety Level; ID, identification.

**Figure 2:**
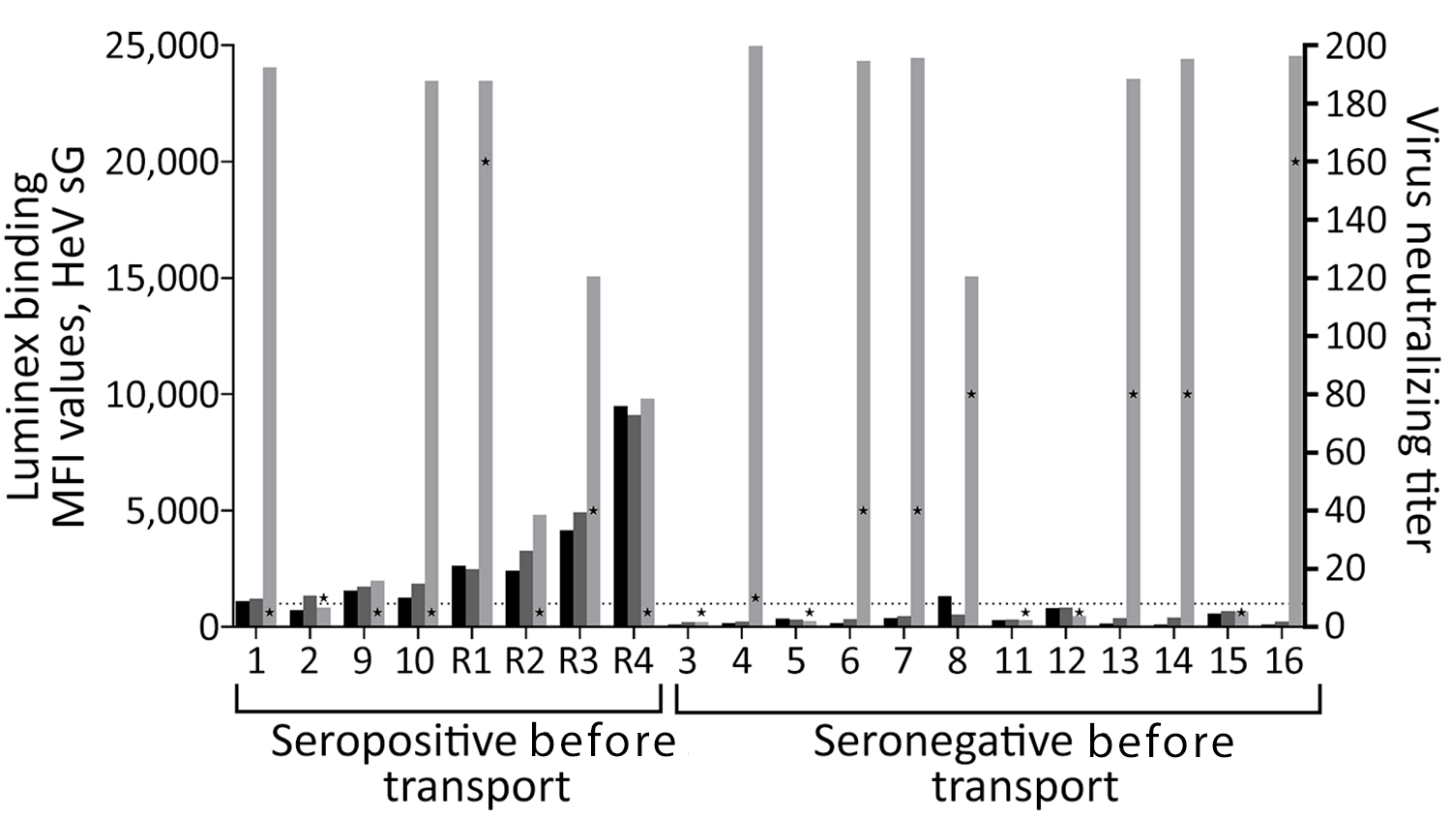
HeV serology of 20 flying foxes following arrival at Australian Centre for Disease Preparedness (ACDP) for a study of natural Hendra virus infections in captive Australian black flying foxes, Queensland, Australia. Detection of antibodies against HeV sG by an in-house Luminex indirect antibody binding serology assay in 20 flying foxes monitored in Queensland in December 2022 and February 2023 (black bars) and March 2023 (dark gray bars) before transport and 1 day after arrival at ACDP (light gray bars). Luminex data displayed as bars (left y-axis) representing MFI. Virus neutralization tests shown as star symbols (right y-axis). Samples with MFI >1000 (dotted line) considered positive. MFI values classified as high (>10,000), medium (4,000–10,000), low (1,000–4,000), and negative (<1,000). Virus neutralization test results were performed only on samples collected 1 day after arrival at ACDP. Bat identification numbers are indicated on the x-axis. All Luminex serologic testing on samples collected in Queensland and ACDP were performed at ACDP on a Bioplex 200 Array system integrated with Bio-Plex Manager Software 6.2 (Bio-Rad Laboratories, https://www.bio-rad.com). MFI, median fluorescence intensity.

Samples collected 1 day after arrival at ACDP revealed that 11 bats had seroconverted positive to HeV during the 3 weeks between testing in Queensland and transport, including 6 previously seronegative and 5 low-seropositive bats (Figure 2). Nine of the 11 bats that had seroconverted to HeV according to the in-house Luminex assay also had neutralizing antibodies to HeV, with virus neutralization test (VNT) titers ranging from 10 to 160 (Figure 2). Two bats tested PCR-positive for HeVg1 RNA: 1 (bat R1) on day 1 postarrival in oral and rectal swab specimens (cycle threshold [Ct] = 33.9) and urine (Ct = 29) and 1 (bat 4) on day 8 in urine (Ct = 25). Both bats had already seroconverted in the 3 weeks between Queensland testing and arrival at ACDP, with bat R1 increasing from low Luminex seropositivity to a VNT titer of 160, and bat 4 from seronegative to VNT titer of 10 (Figure 2). Urine collected from underneath individual cages tested negative for known zoonotic viruses, except for those from bat 4, which were HeVg1 positive from days 9–14 at ACDP.

**Figure 3:**
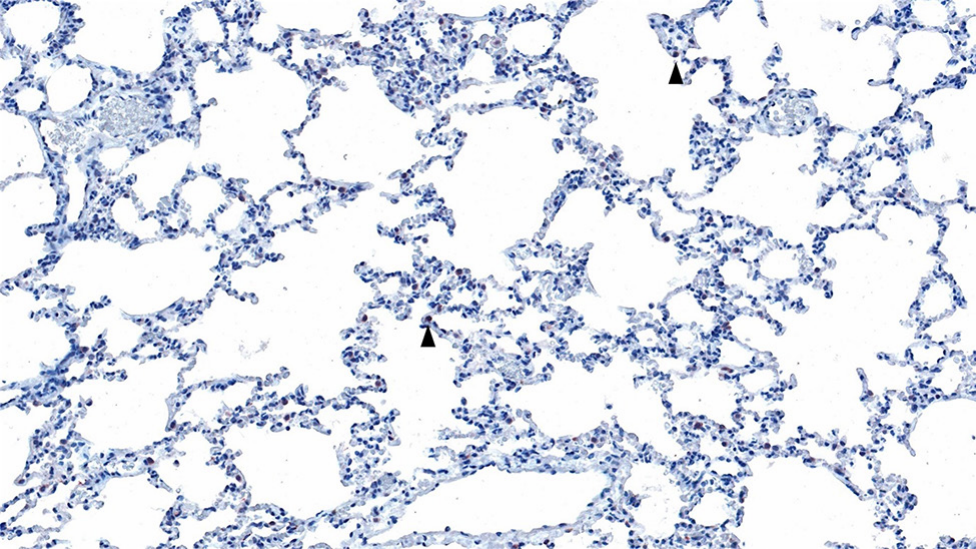
Histopathology of lung from a naturally infected flying fox from a study of natural Hendra virus infections in captive Australian black flying foxes, Queensland, Australia. Immunohistochemistry of bat 4, left caudal lung lobe for Hendra virus (nucleocapsid protein) using a cross-reactive Nipah virus nucleocapsid polyclonal antisera showing scattered interstitial mononuclear cells with finely granular cytoplasmic immunolabelling (arrowhead). Original magnification ×200.

Because of biosafety constraints, we collected no tissues from bat R1, which was housed in a Biosafety Level (BSL) 3 facility (BSL3Z). Bat 4, housed in a BSL-4 facility (BSL4), had HeVg1 RNA detected at necropsy on day 14 in 2 of 14 tissues, the submandibular lymph node (Ct = 39.2) and spleen (Ct = 38.2), and in urine (Ct = 33). Virus isolation was unsuccessful from urine collected on arrival (bats R1 and 4) and from tissues collected from bat 4 at necropsy. Histopathologic analysis of tissues from bat 4 revealed apparent congestion of blood vessels throughout the lungs, as well as the expansion of perivascular tissues by edema. We also noted mild to moderate lymphoid follicular hyperplasia in bat 4, throughout lymphoid organs, but most pronounced in the spleen and mesenteric lymph nodes. No microscopic changes were evident in any other tissues. Viral antigen was only evident in isolated mononuclear cells scattered throughout the pulmonary interstitium (Figure 3).

## Conclusions

This study provides evidence of natural HeV infections and seroconversion in a captive cohort of *P. alecto* bats, including detection of active HeV infections in apparently naive bats and in those with evidence of previous infections. Viral replication in seropositive animals implies that flying foxes undergo cycles of infection and reinfection, and it is thus unlikely that prior infection leads to lifelong immunity in *P. alecto* bats. This finding contrasts with those for some other bat species, which appear to undergo higher rates of seroconversion with immunity to reinfection even after antibodies have waned (*6*,*7*).

The timing of infection—within 3 weeks before transport—is consistent with exposure having occurred in Queensland, either via exposure to HeV excretions from a wild bat outside the enclosure or because of recrudescence followed by horizontal transmission within the colony. Although evidence is insufficient to determine whether viral recrudescence occurred in the bats we studied, increasing evidence suggests that henipaviruses may establish latency and persist in humans and bats (*8*–*11*). Experimentally infected *P. alecto* bats typically begin shedding virus in throat and rectal swab specimens 2–7 days postinfection and in urine 7–19 days postinfection. Experimentally, development of HeV-neutralizing antibody has been inconsistent, but reports have noted low levels detected by 10 days postinfection (*12*–*14*). Thus, we speculate that the 2 flying foxes shedding HeV after arrival were likely infected with HeV ≥1 week before transport. Bat R1, which tested PCR-positive in both oral/rectal swab and urine specimen, likely experienced an active HeV infection within 10 days before transport to ACDP and continued shedding HeV upon arrival. In contrast, bat 4 may have undergone an active infection in Queensland that resolved before transport. Because those 2 bats were housed in separate rooms at ACDP, it is possible that bat 4 was reinfected immediately before transport or underwent viral recrudescence at ACDP.

We detected HeV neutralizing antibodies in 9 of the 11 seroconverted flying foxes upon arrival at ACDP; 3 had titers of 80 and 2 had titers of 160. Prior reports have revealed inconsistent antibody responses in flying foxes experimentally infected with HeV, with around 50% seroconverting with neutralizing titers of 10–80 by 10 days after oronasal inoculation (*14*,*15*). Given the unknown infection histories of bats in our study, higher titers may reflect anamnestic responses to HeV infection or could allude to differences between natural and experimental infections. Further studies will be required to determine the nature of antibody responses to infection and reinfections in flying foxes.

Our findings underscore the complexity of HeV maintenance in bat populations and highlight the need for further studies on immune dynamics, latency, and environmental drivers of recrudescence. Those insights are critical for understanding spillover risk and informing public health strategies.

Appendix Additional information for natural Hendra virus infections in captive Australian black flying foxes, Queensland, Australia.
