# Supplementary material for: Natural Hendra Virus Infections in Captive Australian Black Flying Foxes, Queensland, Australia
Source: Emerg Infect Dis. 2026 Mar;32(3):433–7. doi: 10.3201/eid3203.251350 (PMC13015999; doi:10.3201/eid3203.251350)
Supplement: Appendix — Additional information for natural Hendra virus infections in captive Australian black flying foxes, Queensland, Australia. [file 25-1350-Techapp-s1.pdf]

Article DOI: http://doi.org/10.3201/eid3203.251350

EID cannot ensure accessibility for supplementary materials supplied by authors. Readers who have difficulty accessing supplementary content should contact the authors for assistance.

Natural Hendra Virus Infections in Captive Australian Black Flying Foxes, Queensland, Australia

Appendix

Appendix Table. Luminex indirect antibody binding serology of 39 flying foxes during screening from a study of natural Hendra virus infections in captive Australian black flying foxes, Queensland*

Sex	Age	Bat ID	Location	Dec 2022	Feb 2023	March 2023
F	Adult	3	Beaudesert	100		207
M	Adult	16	Beaudesert	100		235
M	Adult	14	Beaudesert	109		417
F	Sub adult	13	Beaudesert	160		393
M	Adult	6	Beaudesert	173		340
M	Adult	4	Beaudesert	174		234
F	Adult	11	Logan Reserve		304	316
M	Adult	5	Logan Reserve		359	328
M	Adult	7	Beaudesert	393		472
F	Adult	15	Logan Reserve		571	696
M	Sub adult	2	Beaudesert	723		1348
M	Sub adult	12	Beaudesert		822	838
M	Adult	1	Beaudesert	1115		1225
M	Adult	10	Beaudesert	1263		1865
F	Sub adult	8	Beaudesert	1337		537
F	Adult	9	Beaudesert	1569		1727
M	Adult	R2	Beaudesert	2417		3285
M	Juvenile	R1	Beaudesert	2638		2480
F	Adult	R3	Beaudesert	4167		4938
F	Adult	R4	Beaudesert	3435		9100
F	Adult	NA	Beaudesert	2131		
F	Adult	NA	Beaudesert	16886		
F	Juvenile	NA	Beaudesert	25281		
M	Adult	NA	Beaudesert	2638		
F	Adult	NA	Beaudesert	1294		
F	Adult	NA	Beaudesert	2323		
F	Adult	NA	Beaudesert	4264		
M	Adult	NA	Beaudesert	15171		
M	Adult	NA	Beaudesert		304	
M	Juvenile	NA	Beaudesert		822	
M	Adult	NA	Beaudesert	13053		
M	Adult	NA	Beaudesert	2243		
F	Adult	NA	Beaudesert	23217		
M	Adult	NA	Beaudesert	3042		
F	Adult	NA	Beaudesert	9495		
M	Adult	NA	Beaudesert	1110		
M	Juvenile	NA	Beaudesert	1569		
M	Adult	NA	Beaudesert	25236		
M	Adult	NA	Beaudesert	25708		

*All screened individuals were healthy but had minor injuries, predominantly to the wings that prevented their release. Those individuals chosen for transport to ACDP shown in bold and were those having both the lowest HeV serostatus and the best overall condition. Data represented as mean fluorescent intensity (MFI). MFI values were classified as high (>10,000), medium (4,000–10,000), low (1,000–4,000), and negative (<1,000).
